# A scalable fish-school inspired self-assembled particle system for solar-powered water-solute separation

**DOI:** 10.1093/nsr/nwab065

**Published:** 2021-04-21

**Authors:** Ning Xu, Haoran Zhang, Zhenhui Lin, Jinlei Li, Guoliang Liu, Xiuqiang Li, Wei Zhao, Xinzhe Min, Pengcheng Yao, Lin Zhou, Yan Song, Bin Zhu, Shining Zhu, Jia Zhu

**Keywords:** solar-vapor conversion, self-assemble, collective system, water-solute separation

## Abstract

Complete separation of water and solute is the ultimate goal of water treatment, for maximized resource recycling. However, commercialized approaches such as evaporative crystallizers consume a large amount of electricity with a significant carbon footprint, leading to calls for alternative energy-efficient and eco-friendly strategies. Here, inspired by schooling fish, we demonstrate a collective system self-assembled by expanded polystyrene (EPS)-core/graphene oxide (GO)-shell particles, which enables autonomous, efficient and complete water-solute separation powered by sunlight. By taking advantage of surface tension, these tailored particles school together naturally and are bonded as a system to function collectively and coordinatively, to nucleate, grow and output salt crystals continuously and automatically out of even saturated brine, to complete water-solute separation. Solar-vapor conversion efficiency over 90% and salt production rate as high as 0.39 kg m^–2^ h^–1^ are achieved under 1-sun illumination for this system. It reduces the carbon footprint of ∼50 kg for treating 1-ton saturated brine compared with the commercialized approaches.

## Teaser

It is important, although very challenging, to realize eco-friendly water-solute separation with minimized carbon footprint. This paper demonstrates a scalable fish-school inspired self-assembled particle system, which enables autonomous, efficient and complete water-solute separation powered by sunlight.

## INTRODUCTION

The water crisis is among the most severe global challenges, as natural freshwater has been substantially depleted through developments in agriculture and industry [1,2]. Desalination and purification of water is emerging as a useful strategy; however, this is often limited by energy consumption and associated environmental and economic costs. Complete separation of water and solute has been pursued as the ultimate target for water reclaiming and resource recycling [3–5]. However, commercialized approaches such as evaporative crystallizers consume a tremendous amount of energy and generate a large carbon footprint, leading to calls for development of energy-efficient and eco-friendly strategies [6–8].

The solar evaporation approach, which can evaporate water at the surface using solar energy [9–21], has already shown promising prospects in clean water production and water treatment because of its high energy conversion efficiency. For clean water production, elaborately designed absorber and multi-stage devices have been developed, realizing a high water yield [22–25]. For water treatment, the solar evaporation approach also demonstrates initial effort towards water-solute separation [26–32]. However, so far, a scalable pathway for autonomous, continuous and efficient salt production out of high salinity brine has not been realized.

**Figure 1.**
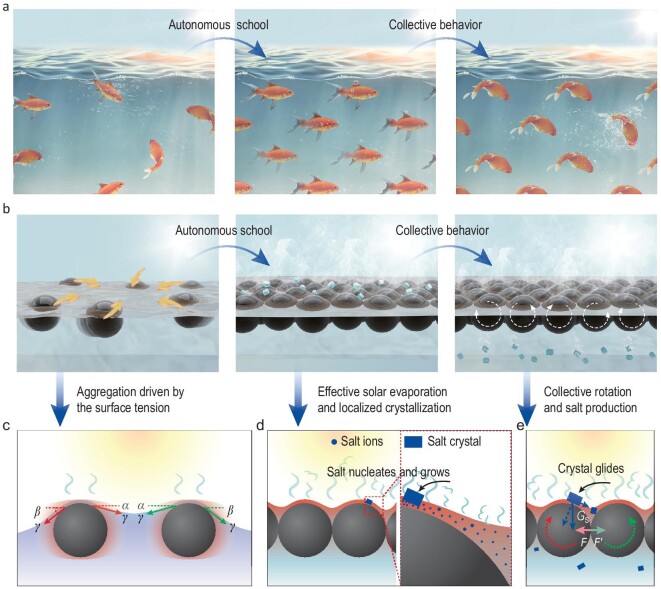
Schematics of interactive particles schooling together by surface tension as a collective system for autonomous solar-powered water-solute separation. (a) Fish school together as a collective system, in which each fish can drive as well as respond to the actions of the neighboring fish. (b) Fish-school inspired collective system self-assembled by particles for solar-powered autonomous water-solute separation. (c) The schooling behavior of the particles driven by surface tension. (d) The solar-thermal conversion process for the aggregated particles. The aggregation is ideal for localizing energy to evaporate water and nucleate salt. When treating brine with saturated concentration, the particle absorbs the light to evaporate the water. This process also nucleates and grows salt locally among the water film on the top of the particle because of the inhomogeneous evaporation, eventually leading to rotation of the particles and glide of salt crystals. (e) Collective behavior of the particle school. All the particles are bonded and interactive as a result of surface tension, in which the rotation of one particle will drive the rotations of adjacent membranes, and consequentially affect the entire system.

To design and construct a scalable and autonomous system for water treatment, one may draw inspiration from fish schooling, one of the most prominent aggregation behaviors and collective intelligence seen in nature (Fig. 1a). While each fish has its own behavior, they tend to school together, to minimize the overall energy of the system [33,34], and to collectively and coordinatively achieve effective functionalities such as defense and foraging [35]. The key to realizing this correlated and collective intelligence is interactions (visual contact, hearing, etc.), by which each fish can drive as well as respond to the actions of the neighboring fish [36].

In this study, surface tension was used as an interactive force among building blocks to form a collective system [36–41]. For surface tension to play a dominant role, expanded polystyrene (EPS)-core/graphene oxide (GO)-shell particles of carefully tailored size, shape and surface chemistry serve as the building blocks (Fig. 1b). These particles can school together naturally and be bonded as a dynamic system to function collectively and coordinatively to enable autonomous water-solute separation powered by sunlight.

Driven by surface tension (Fig. 1c), these particles mimic the behaviors of schooling fish in various aspects. These particles can school together with minimized surface energy. This aggregation behavior is beneficial for thermal localization, and therefore more effective solar evaporation (Fig. 1d). As a result of surface tension, an inhomogeneous brine film (thinner at the top) also forms above the particle. As the brine film at the top of the particle evaporates faster under solar illumination, salt tends to nucleate and grow among the water film at the top of the particles (Fig. 1d). Also, as fish in a school react to the external stimulus, the particle automatically rotates, cleans itself and re-starts the cycle when the as-grown salt crystals break the mechanical balance (Fig. 1e). More interestingly, similar to direct interaction among schooling fish which leads to the coordinating behavior, the strong bonding among these particles as a result of the surface tension leads to a collective and coordinative system (Fig. 1e). The rotation of one particle will drive the rotations of adjacent members.

Altogether the ‘schooling fish’-like interactive particle schools act as an autonomous water-solute separator powered by sunlight, which realizes an energy conversion efficiency above 90% and a salt production rate as high as 0.39 kg m^–2^ h^–1^ under 1-sun illumination. As a result, the scalable and autonomous solar-powered system provides an innovative pathway for complete water-solute separation with minimized power consumption and carbon footprint.

## RESULTS AND DISCUSSION

### Tailored EPS-core/GO-shell particle as the building block

**Figure 2.**
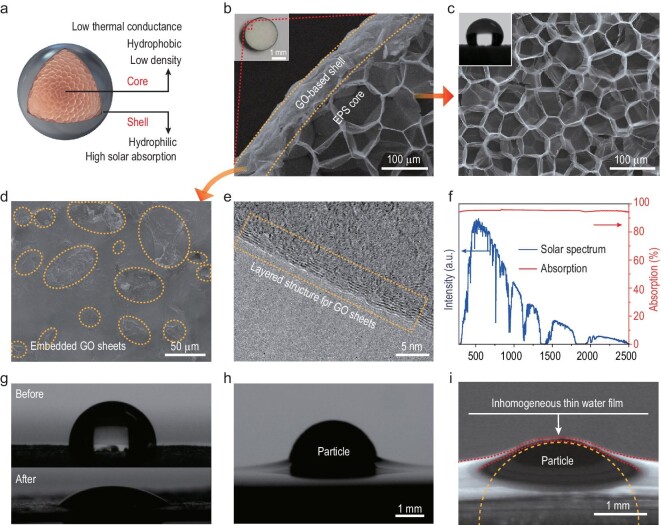
Designs and characterizations of the EPS-core/GO-shell particles as the key building blocks. (a) Design of the EPS foam-core/GO-shell particle. The shell should be hydrophilic with high solar absorption, while the core should be hydrophobic with low density and low thermal conductance. (b) Optical (inset) and scanning electron microscopy (SEM) images show the cross-sectional view of the EPS-core/GO-shell particle. (c) SEM image of the porous EPS foam as the core. The inset is the contact angle of the EPS foam, which confirms its hydrophobicity. (d) SEM photos of the GO-based shell. (e) TEM image of the GO sheets. (f) The absorption spectrum of the GO-based shell. (g) The contact angles of the shell surface before and after the hydrophilic treatment. (h) Photograph of the particle (before the treatment) floating on the water. (i) Photograph of the particle (after the treatment) in water. The particle floats with an inhomogeneous thin water film around it (red dash line) once it is immersed in water. The orange dash line shows the outline of the particle.

As surface tension plays a dominant role in the construction of the collective system and photo-thermal management is critical for efficient solar evaporation, polystyrene (EPS)-core/graphene oxide (GO)-shell particles were carefully tailored to serve as building blocks (Fig. 2a and b). Spherical-shaped particles were chosen, as they provide infinite axes of symmetry, beneficial for scalable self-assembly and autonomous rotation. The diameter of the particles was selected as ∼3 mm in consideration of heat localization and self-cleaning ability (Figs S1 and S2 in the online Supplementary Data). The EPS core is hydrophobic with low density and low thermal conductance, which makes the particle naturally float on water with reduced conductive heat loss. The GO-based shell is hydrophilic with high solar absorption, so that the particle can fully absorb solar energy and be influenced by surface tension. All of these properties are carefully examined below.

Figure 2c shows that the EPS foam as the core is highly porous (with a density of 25 kg m^–3^) and hydrophobic, which is critical to keep the structure floating on the water surface. This porous structure also endows a low thermal conductivity of 0.032 W m^–1^ K^–1^, much lower than that of the water (0.6 W m^–2^ K^–1^), ideal for minimizing conduction loss and enhancing thermal localization (Fig. S3).

The flexible GO-based composite (with ethylene-vinyl acetate (EVA) based binder) was used to form the shell. As shown in Fig. 2d and e, layered GO sheets of micrometer diameter dispersed in the shell. Peaks at ∼1350 cm^–1^ and ∼1590 cm^–1^ of the Raman spectra are consistent with the D band and G band of the GO (Fig. S4), respectively. The GO-based shell demonstrates broadband and efficient absorption (∼95% weighted by AM 1.5G solar spectrum) through the reduced reflection and transmission (Fig. 2g).

As hydrophilicity is critical to generate a thin water film around the particles and provide water supply, the GO shell was treated with nitric acid to enhance the hydrophilicity (Fig. 2g). Before the treatment, the particle just floats on the water surface, and water cannot be supplied to the entire surface of the particle (Fig. 2h). After the treatment and immersing the particle in water, the particle floated with an inhomogeneous thin water film around it (Fig. 2i). The thin water film with gradient thickness on the top of the particle is vital not only for faster solar evaporation (Fig. S5), but also for the particle schooling localized crystallization, and autonomous water-solute separation, as demonstrated in more detail below.

### Surface tension-induced EPS-core/GO-shell particle school as a collective system

**Figure 3.**
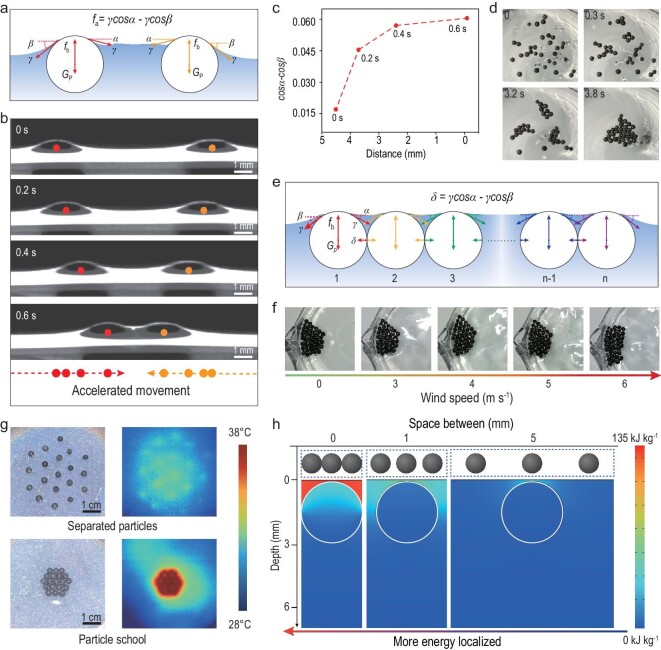
Surface tension-induced EPS-core/GO-shell particle school as a scalable collective system. (a) Mechanical analysis of the encounters of the two particles. *γ* denotes the surface tension between the particle and water; *G*_P_ and *f*_b_ denote the gravity and buoyancy of the particle; *α* and *β* are the slope angles of the water menisci to the adjacent side and the free side for the particle. The attractive force *f*_a_ is calculated to be *γcosα−γcosβ.* (b) Time-lapsed side-view images of the encounters of the two particles. (c) Values of (*cosα−cosβ*) over distance, indicating that the attractive force (*γcosα−γcosβ*) increases with the shortened distance. It also indicates that the process of encounter is an accelerating movement with increased acceleration. (d) Top-view images for the schooling process of the multiple particles. (e) Mechanical analysis of the particle school as a whole system bonded by surface tension. (f) Optical photos of the bonded particle school. It maintains integrity even under strong wind. (g) Optical photos of the particle school and the separated particles, as well as their infrared (IR) images under 1-sun illumination for 10 minutes. The higher surface temperature can be found in the particle school compared with separated particles, indicating stronger heat localization and faster solar evaporation for particle schools. (h) Simulative analysis of internal energy distribution for closely packed particle school (zero space) and hypothetic discrete particles (1 mm and 5 mm apart). Much more energy is localized in the top water film for the closely packed particle school.

Once the tailored particles are put on the water surface, similar to aggregation behavior of fish, the particles school together naturally to form a bonded system by surface tension with minimized surface energy. This can be ascribed to the unbalanced horizontal components of surface tension (*γ*), generated by the asymmetric distorted water film on the particle. For two particles placed near to each other on the water surface, *G*_P_ and *f*_b_ denote the gravity and buoyancy of the particle, and *α* and *β* are defined as the slope angles of the water menisci for the adjacent side and the free side, respectively (Fig. 3a). For vertical direction, there exists a delicate balance of gravity, buoyancy and surface tension (*G*_P_ + *γsinα* + *γsinβ = f*_b_). While for horizontal direction, because of the overlapping and thus elevated water menisci between the two particles, there exists a disparity between *β* and *α* (*β > α*), generating attractive forces *f_a_ = γcosα- γcosβ > *0, which leads to the encounter of the two particles.

Figure 3b demonstrates side-view images of the encounter process for the two particles, as they are placed near on the water surface (the entire process is given in Movie S1). This shows that two particles 4 mm apart encounter within 0.6 seconds. The increased length of the movement over the same interval (two dash arrows) suggests accelerating movements for the two particles. It was found that the value of (*cosα-cosβ*), and therefore the attractive force (*γcosα-γcosβ*), increases over time as two particles move towards each other (Fig. 3c). This indicates that the process of encounter is an accelerating movement with increased acceleration, which means particles over a large distance can encounter in a short period of time.

This increased attractive force for shorter distance dictates that for the multi-particle system, the schooling process always preferentially occurs first to particles located closer because of stronger attraction forces, and the rest of the particles school together subsequently. A schooling process for multiple particles is shown in Fig. 3d and Movie S2. Particles with closer intervals school into small clusters first, and then aggregate into a whole system. It is clear that schooling happens very quickly, as particles that spread on a water surface with an area of ∼5×5 cm school into a correlated system within 4 seconds. The spontaneous and quick aggregation behavior of the particles is ideal for constructing large-scale (over 100 m^2^) particle schools for practical applications (Fig. S6).

Once these particles school together quickly, the particles are bonded as a collective system by surface tension, which is shown as the bonding force *δ* (*δ = γcosα − γcosβ*) in Fig. 3e. As a result of strong bonding, the particle school is capable of maintaining integrity even under wind of 6 m s^–1^ (Fig. 3f and Movie S3). This is ideal for practical applications. The bonded particle schools are also beneficial for heat localization compared to discrete particles. As shown in Fig. 3g, the bonded particle school has a higher working temperature (∼36.5ºC) compared with that of the separated particle (∼32ºC) under 1-sun illumination for 10 minutes, which indicates stronger heat localization for the particle school. The internal energy distributions of the infinite closely bonded particle school (zero space) and hypothetic infinite discrete particles (particles 1 mm apart and particles 5 mm apart) were also compared using COMSOL simulation (Fig. 3h). It was found that much more energy is localized in the top water film for the closely bonded particle school compared with discrete particles, beneficial for efficient solar evaporation and salt precipitation (Fig. S7).

### Autonomous salt production and self-cleaning of the particle school

**Figure 4.**
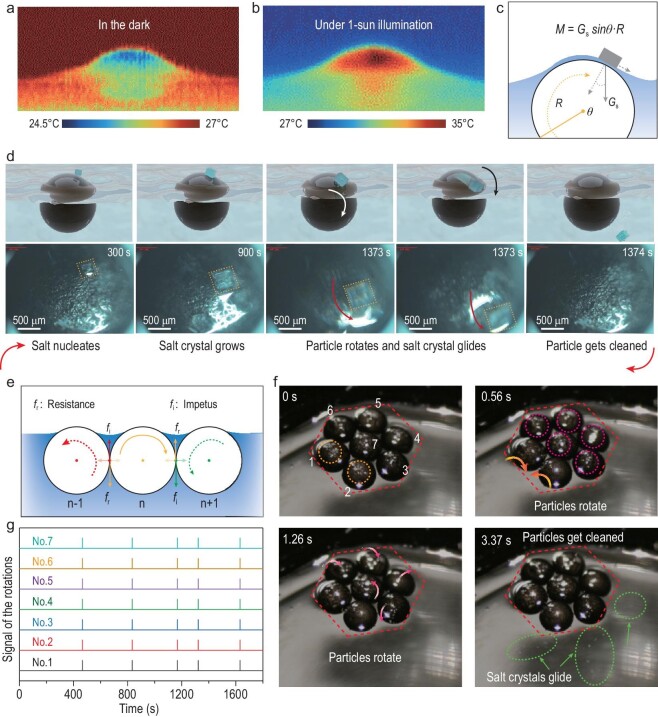
Autonomous salt production as well as self-cleaning for the particle school. IR images of the floating particle in the darkness (a) and under 1-sun illumination (b). (c) Schematic of loaded torque on the particle during the growth of the salt crystal on the particle surface. (d) Schematics and optical microscope photographs (five times amplification) of the repeated process of salt production as well as self-cleaning: nucleation and growth of the salt crystal, rotation of particle, the glide of the salt crystal and reset of the process. (e) Mechanical analysis of the correlated rotations in this system. (f) Optical photos for the process of the correlated rotations. A hepta-particle (heptamer) system is chosen as an example for studying the correlated dynamic rotations. The orange and violet dash circles denote the salt crystals on the particles, which glide after the rotations (denoted by orange and violet arrows). Salt crystals finally glide into brine water, as shown by the green arrows and circles. (g) Signals of the rotations for each member (particle) during the 30 minutes solar evaporation out of saturated brine.

As demonstrated previously (Fig. 2i), there exists a thin water film above the tailored EPS foam-core/GO-shell particle because of the surface tension. This water film is inhomogeneous, with the thinner part located on the top. It is clear that (Fig. 4a), in the dark (without sunlight), the top part of the particle has a lower temperature, indicating that faster water evaporation occurs at the top. Under 1-sun illumination, the top part of the particle exhibits a higher temperature, while the above thin water film maintains a homogeneous temperature, which is realized via the stronger evaporation of water at the top position (Fig. 4b). Thus, as high evaporation always happens at the top part, it is also the easiest for this part to reach the critical crystallization concentration during continuous solar water treatment.

As the salt nucleates and grows among the water film at the top of the particle, it loads torque *M* (*M = G*_s_*sinθR, G*_s_ denotes the gravity of the salt crystal; *θ* denotes the location of the mass center of the precipitating salt; *R* denotes the radius of the particle) on the particle (loading through the force of friction between the particle and the salt crystals) (Fig. 4c). This torque breaks the balance of the particle, leading to the rotation. Because of the water film, the grown salt crystals stay on rather than stick to the particle [31], so that they easily glide after the rotation, making the particle self-cleaning (Fig. S8). The whole process was constantly repeated during the treatment. The entire dynamic behavior of the particle under sunlight was carefully observed through a microscope, as shown in Fig. 4d (from Movie S4). Firstly, liquid phase nucleation was observed at the top of the particle as evaporation of saturated brine continues. In the following ∼20 minutes, the nucleus grew into the salt crystal gradually, until it broke the mechanical balance (typically when the salt crystal grows into the scale of ∼0.2 mg) and led to a quick rotation (within 1 second) of the particle. Immediately after, the salt crystal glided and fell to the bottom of the container, and a new cycle re-started.

Interestingly, just like the collective behavior in a fish school, in which each fish can drive as well as respond to the actions of the neighboring fish, the rotation of each particle (as a result of torque by the salt crystal) can induce correlative rotations of its surrounding counterparts, making the whole system simultaneous self-cleaned. A mechanical analysis of this process is provided in Fig. 4e. As the particle No.n autonomously rotates (driven by the precipitating salt crystals), the frictions among particles (resembling the lateral line, visual contact, or hearing of fish) are the impetus (*f*_i_) to enable rotations of neighboring particles (No.n − 1 and No.n + 1), making this particle school behave interactively and correlatively.

The collective dynamic behaviors (rotations and crystals glide) under 1-sun illumination were observed in a hepta-particle (heptamer) school as an example (recorded by a high-speed camera; see Movie S5 for the whole process). For clear demonstration, the members were numbered from No.1 to No.7. As shown in Fig. 4f, rotations of members No.1 and No.2 occurred simultaneously (within 0.56 seconds), as indicated by the orange dash-circles and arrows. The members No.3, No.4, No.5, No.6 and No.7 rotated subsequently (within 1.26 seconds), as indicated by the violet dash-circles and arrows. Salt crystals glided into brine water right after, as denoted by the green arrows and circles, making the particle self-cleaning. Thus, within a few seconds, the correlative rotations led to the cleaning of salt crystals off the surfaces of particles.

Collective rotations and consequent self-cleaning processes were repeated during long-term solar treatment of the saturated brine (see the dynamic process in Movie S6). The rotation events for each member were recorded and are demonstrated in Fig. 4g. It can be observed that all the members rotate and are self-cleaned simultaneously because of their correlations in this heptamer school (Movie S7). The intervals of the adjacent rotations for the heptamer school range from 2.5 to 8 minutes. It was expected that the frequency of the autonomous rotations would be determined by the speed and position of the salt crystal growth for the correlated system. Higher illumination would lead to faster evaporation, more rapid growth of salt crystals and more frequent collective rotations of the particle school. This was confirmed by observing more frequent rotation of the heptamer school (seven times per half hour) under higher illumination (2-sun, Fig. S9 and Movie S8) compared to that under 1-sun illumination (five times per half hour). This autonomous and collective rotation behavior for salt production and self-cleaning of the particle schools is highly scalable, as shown in Movie S9.

### Performance of solar water-solute separation for the particle schools

**Figure 5.**
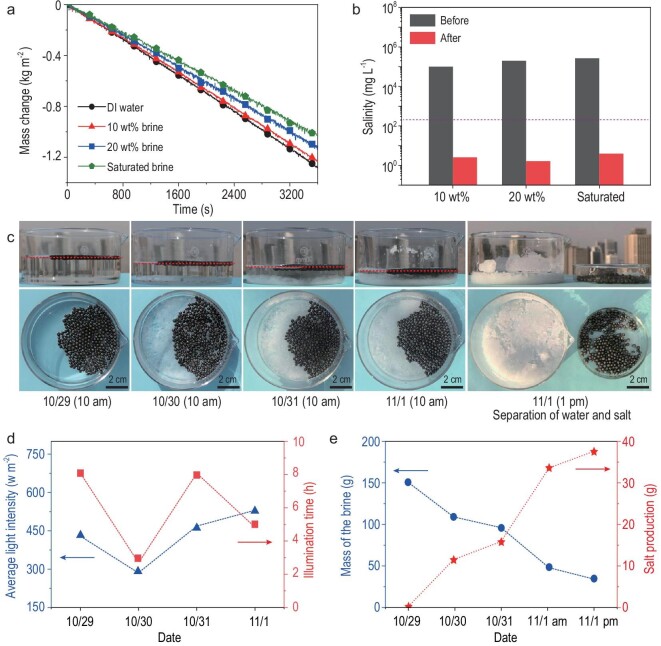
Performance of the autonomous solar water-solute separation for the EPS-core/GO-shell particle schools. (a) Mass change curves of the different water resources (DI water, 10% brine, 20% brine and saturated brine) over time under 1-sun illumination. (b) The salinities of different water resources before and after solar treatment. The dash violet line refers to the WHO standards for drinkable water. (c) The time-dependent photographs with side-view and top-view of the particle schools for outdoor solar water treatment of saturated brine. Water and solute are totally separated after 4-day illumination. (d) The light intensity and illumination hour during 4-day water treatment. (e) Mass changes of the saturated brine and salt production over 4-day water treatment.

The EPS-core/GO-shell particle schools perform well for treating water resources under 1-sun illumination (Fig. 5a). The efficiency of solar-vapor conversion and solar evaporation rate of the deionized (DI) water for the system were calculated to be 90.4% and 1.33 kg m^–2^ h^–1^. The solar evaporation rates of 10 wt% brine, 20 wt% brine and saturated brine were also calculated to be 1.26 kg m^–2^ h^–1^, 1.17 kg m^–2^ h^–1^ and 1.09 kg m^–2^ h^–1^, and the salt production rate reached 0.39 kg m^–2^ when treating saturated brine (Fig. S7). A performance comparison of saturated brine treatment with and without particle schools is demonstrated in Fig. S10. It was found that the salinities for the purified water were all significantly decreased by orders of magnitude (Fig. 5b), meeting the drinking water standards of the World Health Organization (WHO) [42]. The real seawater (collected from the Bohai Sea, China, with an average salinity of ∼1 wt%) can also be well purified (Fig. S11). Using this solar-powered approach, the system reduces electricity of ∼60 kWh and the carbon footprint of ∼50 kg for treating saturated brine of 1 ton compared with a commercialized electricity-powered brine crystallizer [6–8].

Through its unique design, the particle school system maintained stable performance during long-term treatment of the saturated brine thanks to its self-cleaning ability. This is different from a conventional absorber, in which performance degrades as a result of salt clogging (Fig. S12). More interestingly, the particle school system realizes complete solar water-solute separation when treating highly concentrated brine. We performed an outdoor experiment with brine having an initial salinity of 25 wt% (nearly saturated concentration). As the water evaporated out of the saturated brine progressively, the water surface together with the particle school moved downward quickly. Salt crystals were produced and glided to the bottom of the container, while the surfaces of particles remained clean. At the end of the brine treatment, particles can be easily recycled, and the water and salt were completely separated (Fig. 5c). The weather conditions (illumination intensity and duration) for the treatment over time are recorded in Fig. 5d and Table S1. The mass change of the brine and the calculated production of salt over time are also demonstrated in Fig. 5e.

## CONCLUSION

In summary, we report that fish-school inspired EPS-core/GO-shell particles can function collectively and coordinatively as an autonomous solar water-solute separator, as a result of surface tension-dominated interactive forces. This scalable interactive system powered by sunlight, formed by particles with autonomous self-cleaning property, enables nucleation, growth and output of salt crystals out of saturated brine under sunlight, to the complete separation of water and solute. The system realizes solar-vapor conversion efficiency of over 90% and salt production rate as high as 0.39 kg m^–2^ h^–1^ under 1-sun illumination. It reduces the carbon footprint of ∼50 kg for treating 1 ton saturated brine compared with the commercialized approaches. Therefore, it is expected that this autonomous solar water-solute separator formed by a self-driven bottom-up approach will not only enable various solar-powered implications such as water treatment, crystal growth/production and resource recovery but also inspire pathways for building other smart energy materials, devices and systems.

## METHODS

### Fabrication of the EPS-core/GO-shell particle

A commercial spherical EPS foam (foaming capacity of 4000 wt%) with a diameter of ∼3 mm was used as the core. GO sheets of micrometer diameter were coated onto the EPS foam particles with EVA-based binder to form the EPS foam-core/GO-shell particles. The GO-shell was put into a nitric acid solution for hydrophilic treatment.

### Experimental setup for solar water treatment

A solar simulator (94043A, Newport) equipped with an optical filter for the standard AM 1.5 G spectrum was used for short-time (1 hour) indoor experiments of solar water treatment. Particles were placed in Dewar when measuring the mass change curves under simulated sunlight. The 1-hour experiments of solar evaporation were conducted with an indoor temperature of ∼29ºC and humidity of ∼50%. A xenon lamp with an optical filter of AM 1.5 G spectrum was used for long-term experiments. The mass change of water was measured by using a high accuracy balance (FA 2004, 0.1 mg in accuracy). The conversion efficiency was calculated as = $\dot{m}{h_{{\rm{lv}}}}$/${P_{{\rm{in}}}}$, where $\dot{m}$ is the mass flux of the vapor (mass change per time and per absorption area), ${h_{{\rm{lv}}}}$ denotes the liquid-vapor phase change enthalpy (2450 kJ kg^–1^) and ${P_{{\rm{in}}}}$ is the received power density of solar illumination(1 kW m^–2^) [43]. The outdoor experiments were conducted on the top floor of the Science Building at Nanjing University, China.

### Characterizations

The microscopic structures of the particles were characterized by scanning electron microscopy (TESCAN MIRA3). The absorption spectra of the shell of the particle were measured using UV/vis spectroscopy (UV-3600, Shimadzu) attached with an integrating sphere (ISR-3100). A surface tension-contact angle meter (GBX Digidrop) was used to test the water contact angle of the shell and core for the particle. The thermal conductivity of the core was measured by Hot Disk TPS 2500S. Raman measurements were carried out using a Renishaw inVia Raman microscope. The laser excitation wavelength is 514 nm. The concentrations of cations were monitored by virtue of ICP-OES (PerkinElmer Instruments, PTIMA 5300 DV). The photos and videos of the rotation for one single particle in the system were recorded by the optical microscope (Eclipse LV100D, Nikon). The high-speed videos of the collective dynamic behavior for the particle-assembled system were captured by the high-speed camera (Qianyanlang 5F01C). The video of the long-term collective dynamic was captured by the camera of a smartphone. The photos of the water treatment pond were captured by the unmanned aerial vehicle (phantom 4 advanced). To characterize the heat behavior of the separated particles, we used thin steel wire for separating and fixing the particles, as the particles tend to aggregate together once they are put into the water. The IR images were captured by Fluke Tix 580.

## Supplementary Material

nwab065_Supplemental_Files
