# Supplementary material for: A scalable fish-school inspired self-assembled particle system for solar-powered water-solute separation
Source: Natl Sci Rev. 2021 Apr 21;8(10):nwab065. doi: 10.1093/nsr/nwab065 (PMC8566183; doi:10.1093/nsr/nwab065)
Supplement: nwab065_Supplemental_Files [file nwab065_supplemental_files.zip › Supplementary_data.docx]

Supplementary Information for

**A scalable fish-school inspired self-assembled particles system**

**for solar-powered water-solute separation**

Ning Xu^†^, Haoran Zhang^†^, Zhenhui Lin, Jinlei Li, Guoliang Liu, Xiuqiang Li, Wei Zhao, Xinzhe Min, Pengcheng Yao, Lin Zhou, Yan Song, Bin Zhu, Shining, Zhu, Jia Zhu*

National Laboratory of Solid State Microstructures, College of Engineering and Applied Sciences, Jiangsu Key Laboratory of Artificial Functional Materials, Nanjing University, Nanjing 210093, P. R. China.

†These authors contributed equally to this work

*E-mail: [jiazhu@nju.edu.cn](mailto:jiazhu@nju.edu.cn)

**Content List**

**Note S1.** Modeling for the energy distributions of the particle school.

Figure S1. Simulated energy localization of the particle schools with different diameters of particles.

Figure S2. Degradation of the self-cleaning property during solar brine treatment for the particles with diameters of 6-8 mm.

**Figure S3.** Simulated energy localization of the particle schools with different thermal conductivities of the cores.

**Figure S4.** The Raman spectrum of the GO-based shell.

**Figure S5.** Mass change curves of pure water with particle schools before and after nitrification treatment under 1-sun illumination.

**Figure S6.** Particle schools with a large scale for practical application.

**Figure S7.** The evaporation rates and salt production rate for the particle schools when treating different brine sources.

**Figure S8.** Schematic of the salt precipitation process when treating high salinity brine under sunlight for conventional absorber (a) and the particles system as the absorber (b).

**Figure S9.** Rotations (as signals) for the heptamer school under 2-sun illumination.

**Figure S10.** Performance comparison of saturated brine treatment with and without particle schools.

**Figure S11.** Ion concentrations of seawater and collected water, as well as the photo of the water collection device.

**Figure S12.** Long-term solar water treatment of the saturated brine for the particle school system and the conventional absorber.

**Table S1.** The real-time intensities of solar illumination for outdoor experiments.

**Movie S1.** The encounter process for the two particles.

**Movie S2.** The schooling process for multiple particles.

**Movie S3.** The bonded particle school keeps integrity even under the wind of 6 m s^-1^.

**Movie S4.** Autonomous salt production as well as self-cleaning for the single particle when treating saturated brine.

**Movie S5.** The collective rotations and self-cleaning processes of the heptamer school when treating saturated brine under 1-sun illumination.

**Movie S6.** The dynamic behavior of the heptamer school during the long-term saturated brine treatment under 1-sun illumination.

**Movie S7.** The repeated collective rotations and self-cleaning processes of the heptamer school when treating saturated brine under 1-sun illumination.

**Movie S8.** The dynamic behavior of the heptamer school during the long-term saturated brine treatment under 2-sun illumination.

**Movie S9.** The dynamic behavior of particle school with hundreds of members during the long-term saturated brine treatment under 1-sun illumination.

**Modeling for the energy distributions of the particle school.**

We use the COMSOL Multiphysics® software simulation to build a two-dimensional model for analyzing the energy behaviors of the particles. The heat transfer module is used for this simulation.

Particles are regarded as suspending in the top layer of the water (a thickness of 10 mm) due to the surface tension to simplify the model. The initial temperature of the system (particles and water) is set to be 293.15 K. The density of the particle is 45 kg m^-3^; the thermal conductivity of the particle is 0.032 W m^-1^ K^-1^; heat capacity at constant pressure is 1500 J kg^-1^ K^-1^; the emissivity is 1; the convection coefficient is 5 W m^-2^ K^-1^; coefficient of blackbody radiation is 5.76×10^-8^ W m^-2^ K^-4^;

We use periodic boundaries at both sides to simulate the infinite closely packed particle school and hypothetic discrete particles. The input energy ($q_{0}=$1000 W m^-2^) is added to the upper surface of the particle. The evaporation process is out of consideration in this simulation. The thermal conduction, convection, and radiation are taken into consideration for the system. Here we obtain the internal energy distributions after 1min illumination for demonstrating the heat localization behaviors of the particle schools with diameters of 0.03, 0.3, and 3 mm, and the hypothetic discrete particles with diameters of 3 mm.

We also change the thermal conductivity of the particles to be 0.1, 0.5, 1 W m^-1^ K^-1^ to simulate the energy localization performances of the particle schools with different thermal conductivities of the cores.


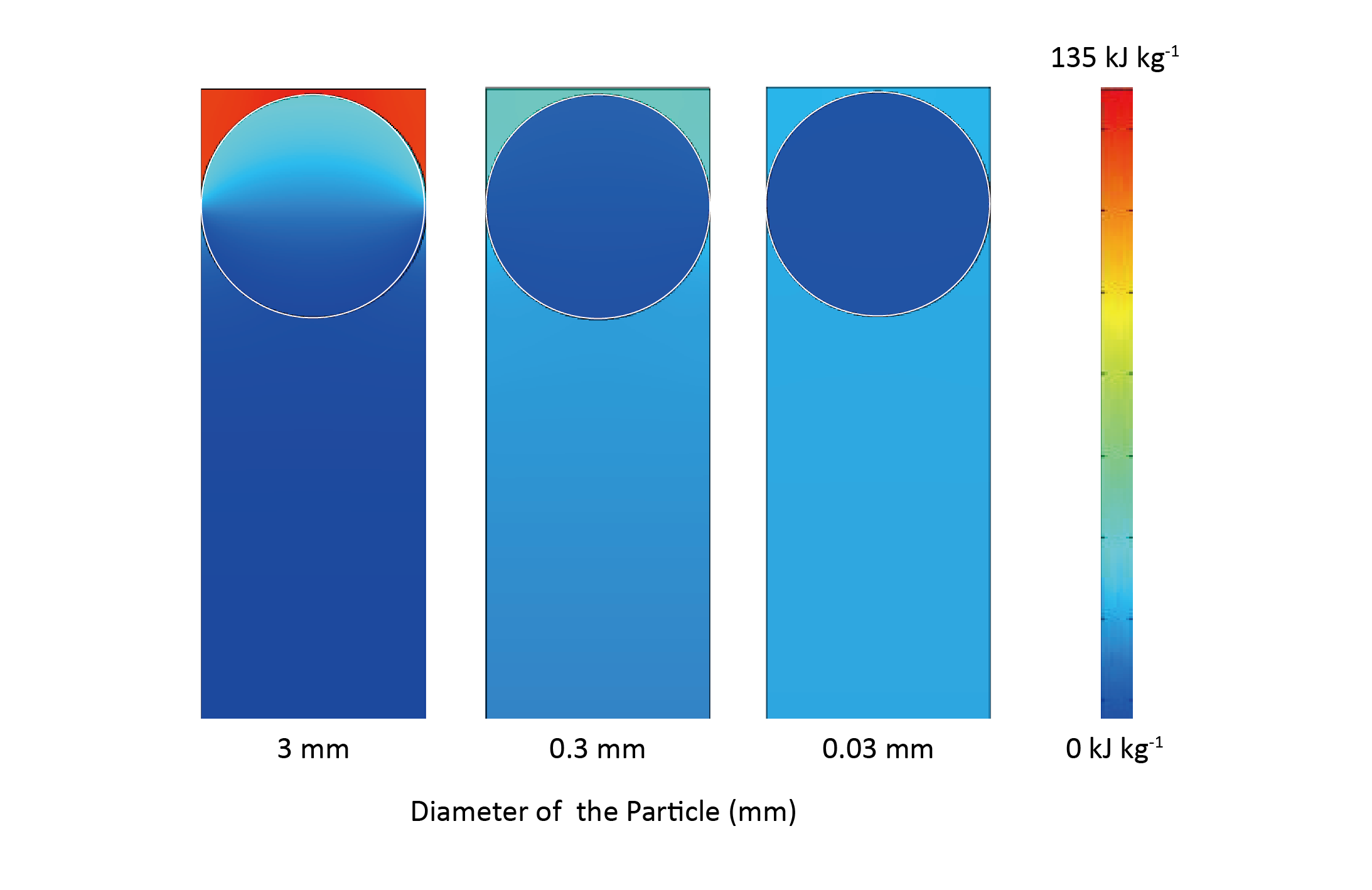


**Figure S1.** Simulated energy localization of the particle schools with different diameters of particles (3 mm, 0.3 mm, and 0.03 mm). Simulated internal energy for each unit in schools with different diameters after 1-minute illumination (1 kW m^-2^ h^-1^). It indicates that the particle schools with a diameter of 3 mm have a stronger solar-thermal localization.


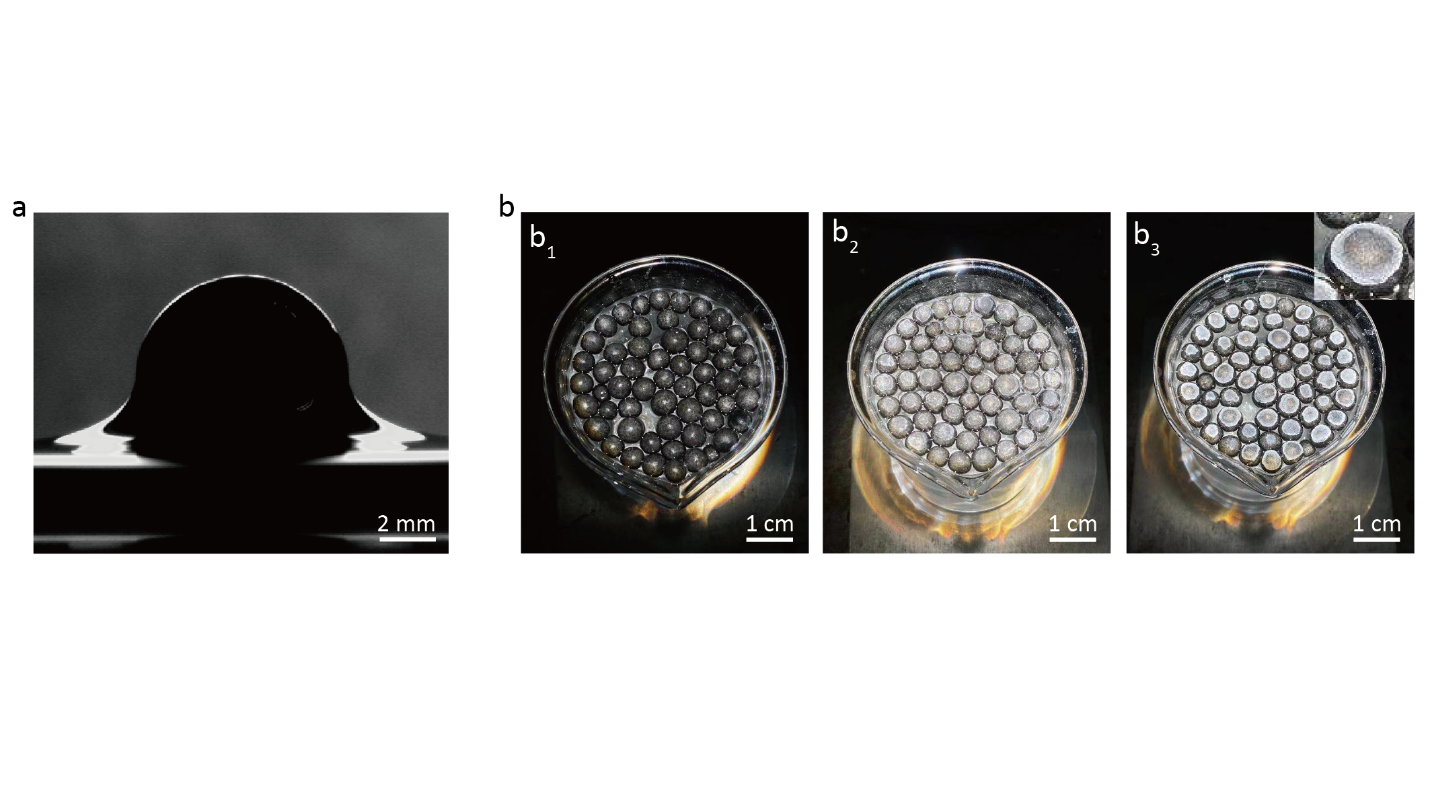


**Figure S2.** Degradation of the self-cleaning property during solar brine treatment for the particles with diameters of 6-8 mm. (a) The thin water film around the particle with a diameter of ~8 mm is incomplete when the particle floats on the water. (b) The photos in order, from (b_1_) to (b_3_), correspond to t=0, 10, and 30 min after the light is on. It is observed that the salt precipitates and grows at the interface of air-water-solid (as the inset in b_3_ shows), and no rotation and self-cleaning behavior occurs during the solar brine treatment.

*
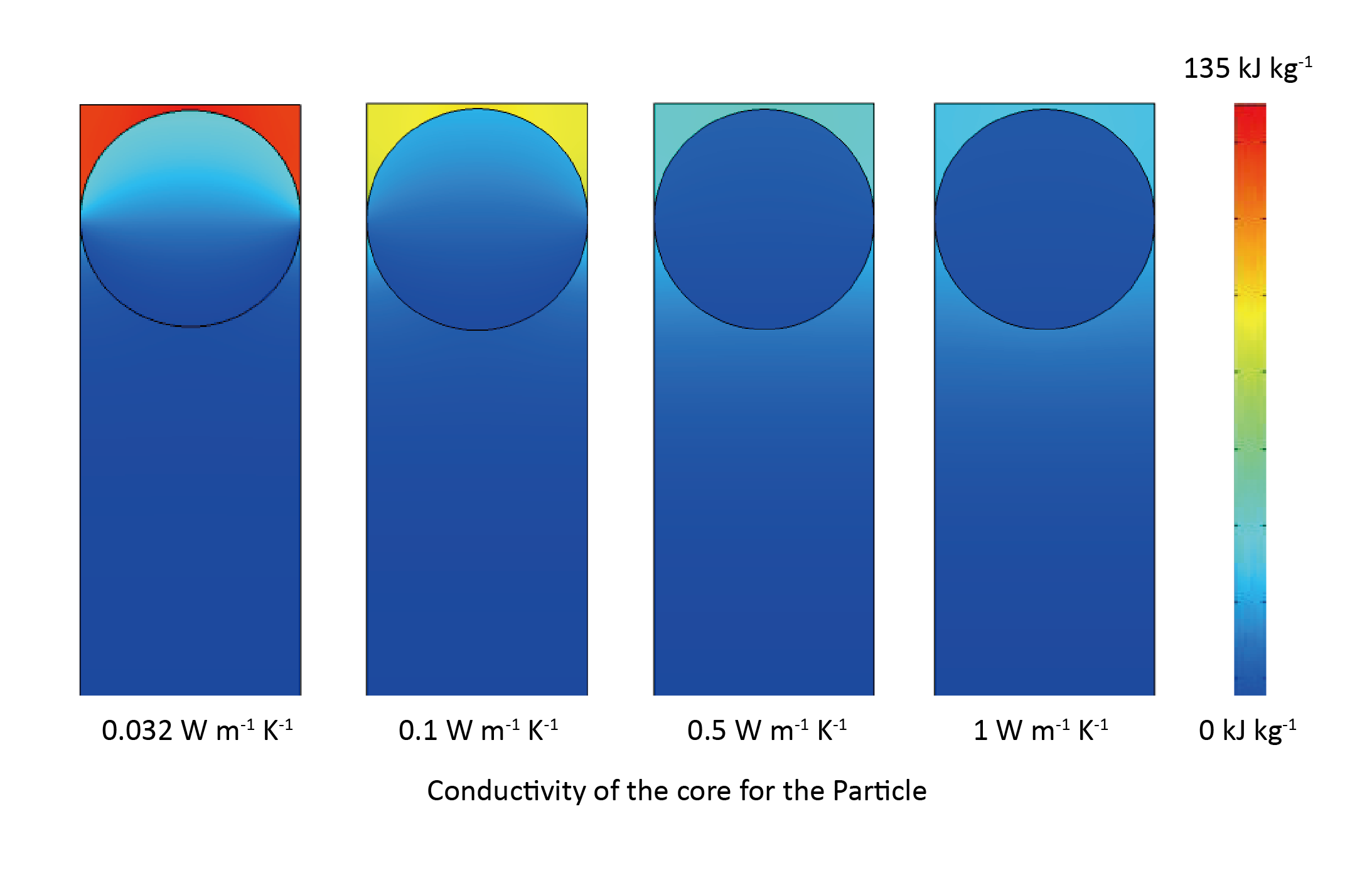
*

**Figure S3.** Simulated energy localization behaviors of the particle schools with different thermal conductivities of the cores.


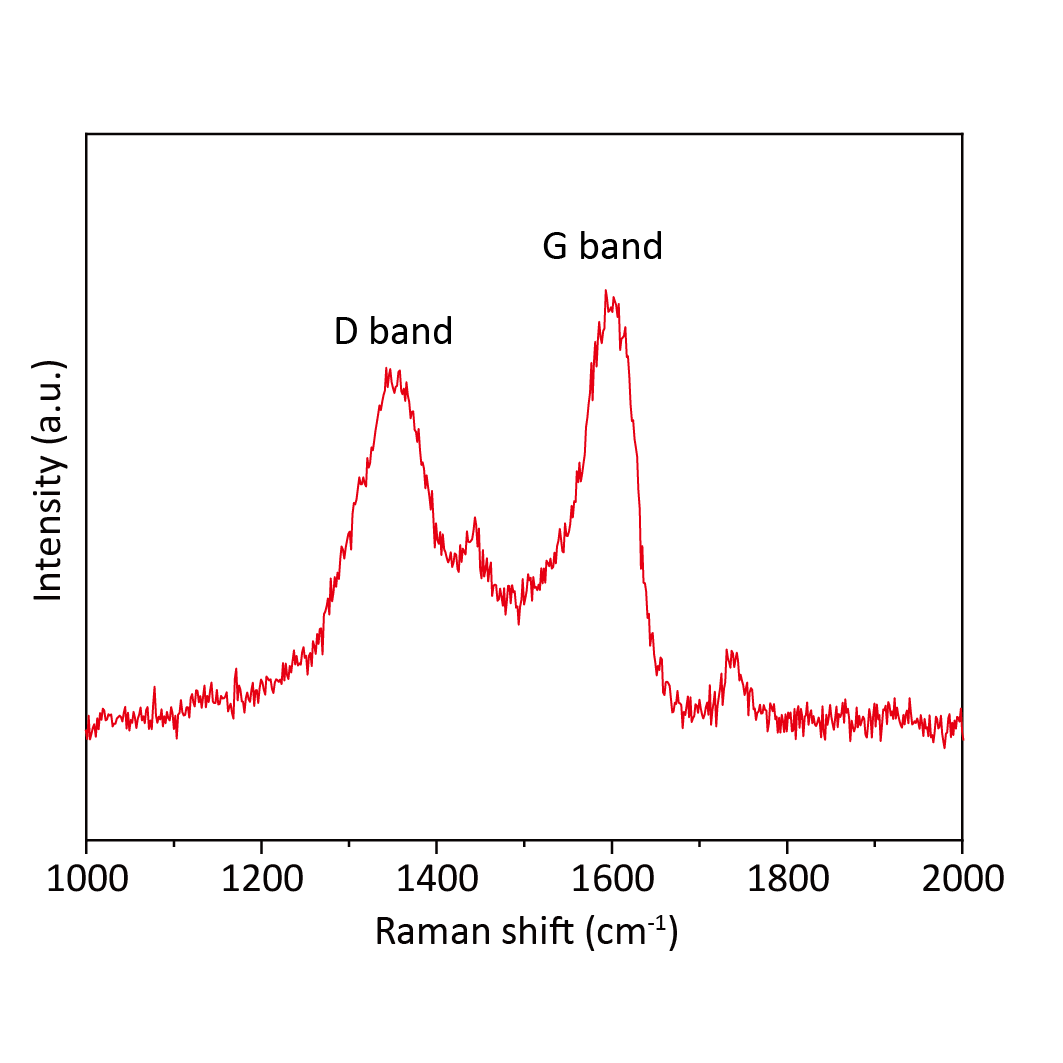


**Figure S4.** The Raman spectrum of the GO-based shell. Peaks at ~1350 cm^-1^ and ~1590 cm^-1^ of the Raman spectra are consistent with the D band and G band of the graphene oxide.

*
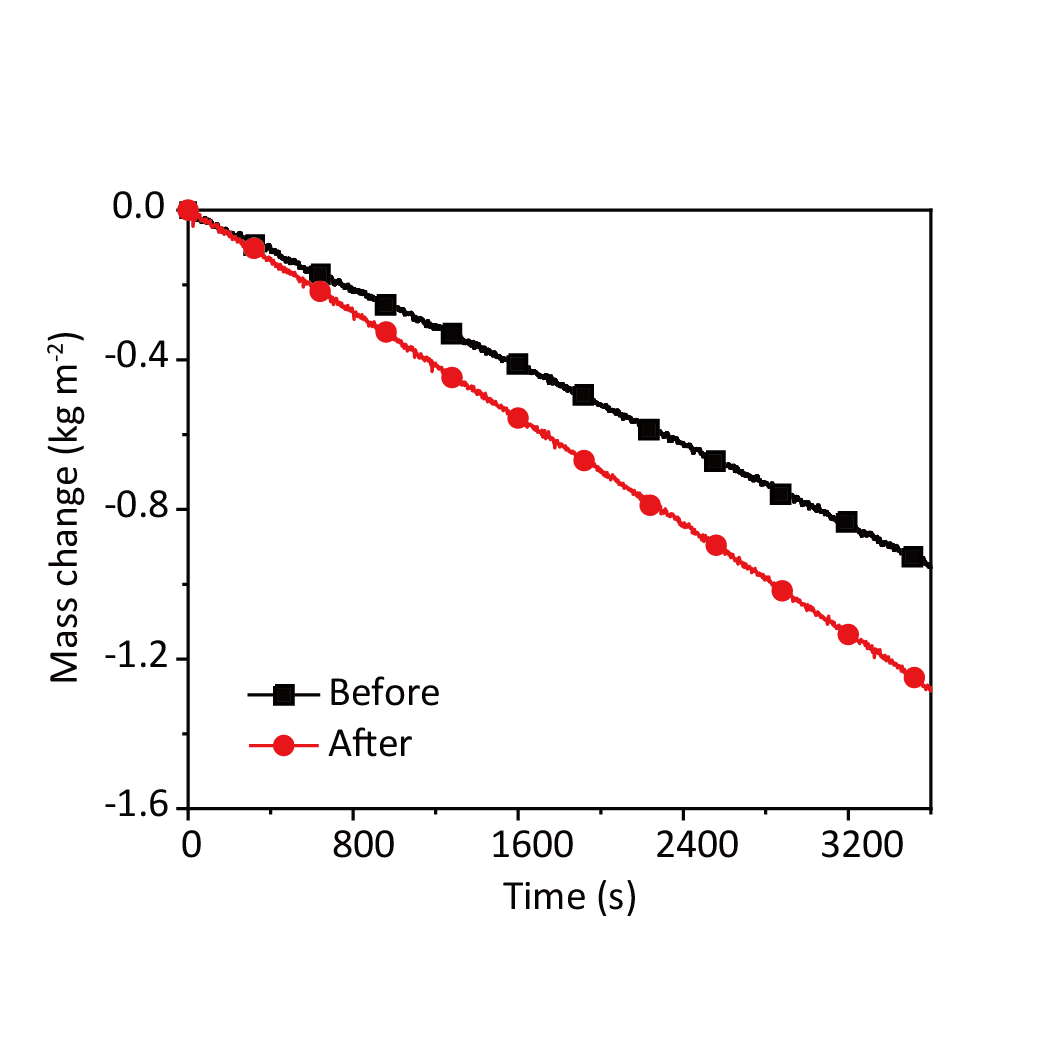
*

**Figure S5.** Mass change curves of pure water with particle schools before and after hydrophilic treatment under 1-sun illumination. It shows that the particle schools after the treatment exhibit faster solar evaporation compared with those before the treatment.


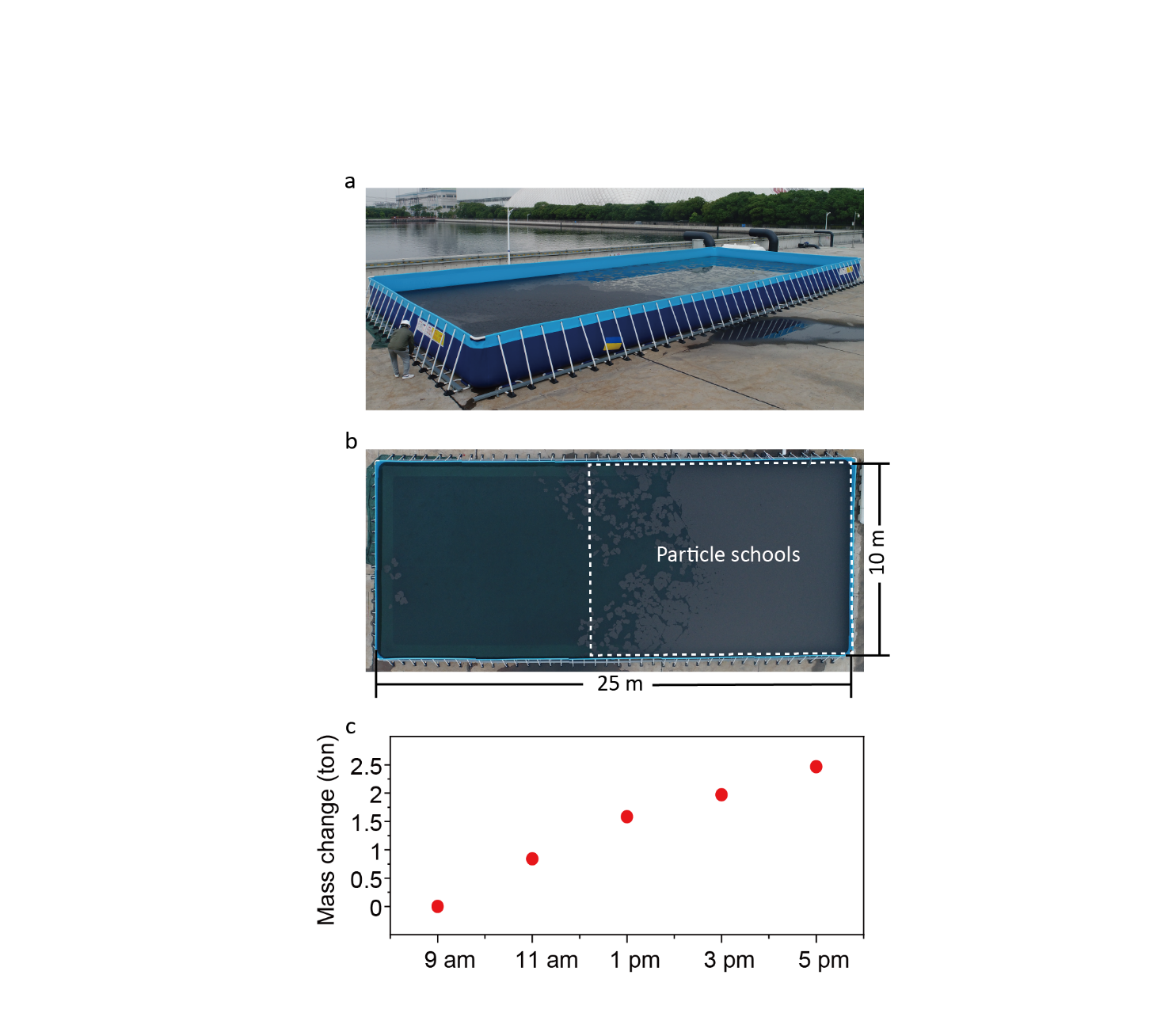


**Figure S6.** Particle schools with a large scale (>100 m^-2^) for practical application. The side-view (a) and top-view (b) photos of the water treatment pond (25 m ×10 m) with particle schools captured by the [unmanned](javascript:;) [aerial](javascript:;) [vehicle](javascript:;). (c) Evaporation performance of the pond. The water of ~ 2.5 tons can be treated during day time (from 9 am to 5 pm).


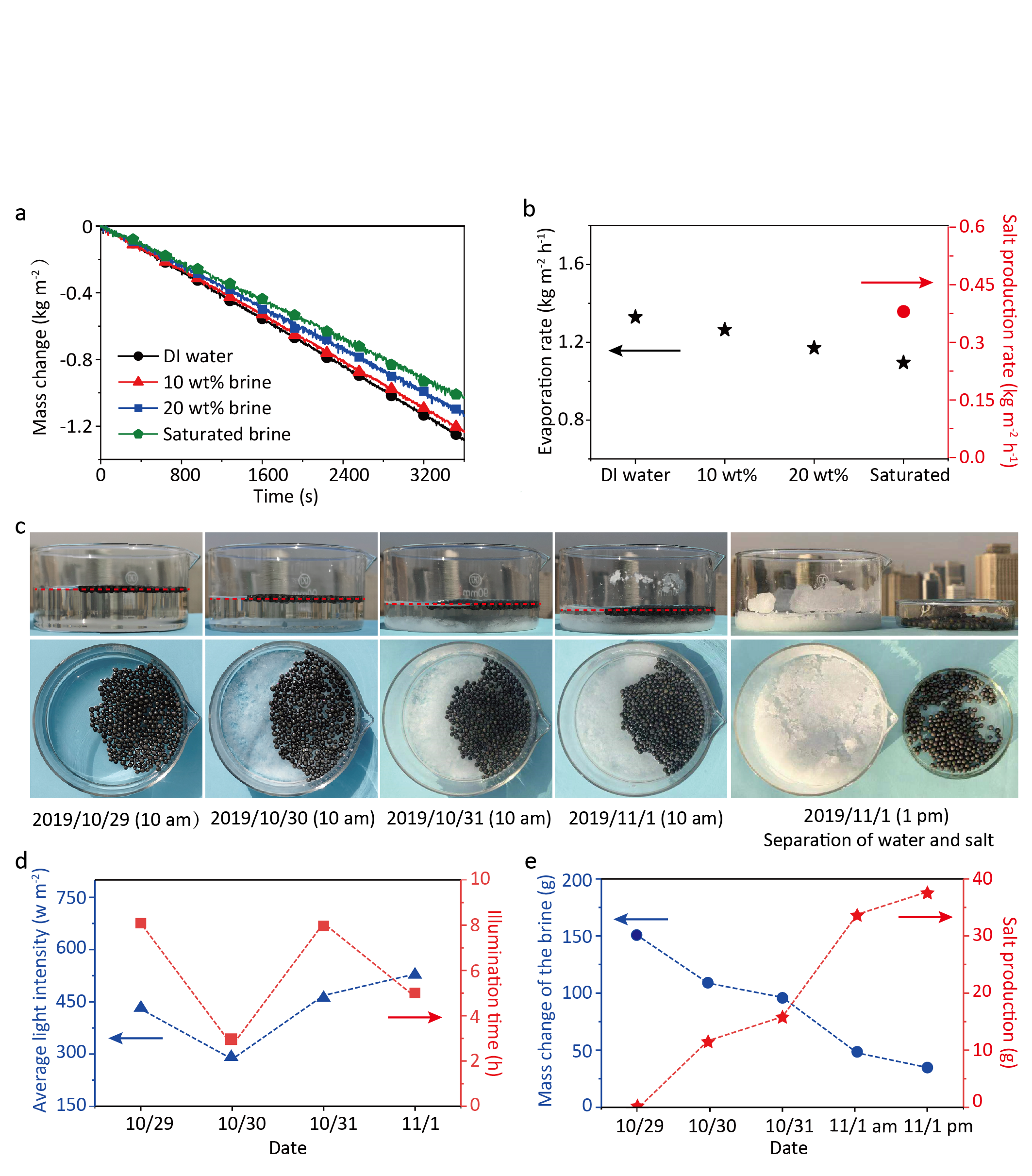


**Figure S7.** The evaporation rates and salt production rate for the particle schools when treating different brine sources.


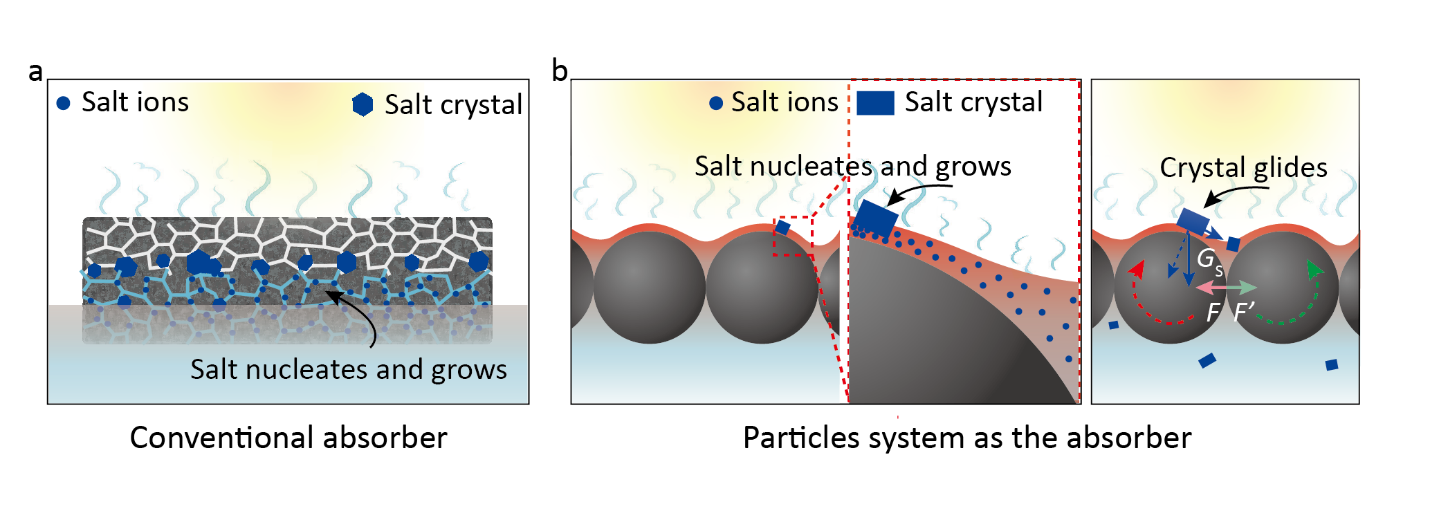


**Figure S8.** Schematic of the salt precipitation process when treating high salinity brine under sunlight for conventional absorber (a) and the particles system as the absorber (b). For conventional absorber, the salt precipitate in the water path among the absorber, making the salt crystals grow and accumulate in the pores of the conventional absorber. For the particle system as the absorber, there exists a thin water film on the particle due to the surface tension, and the salt nucleates and grows among this water film. Thanks to the water film, the grown salt crystals just stay on rather than stick to the particle [1]. Thus, as the particle rotates due to the toque loaded by the salt crystals (loading through the force of friction between the particle and the salt crystals), the salt crystals can easily glide, making the particle self-cleaned.


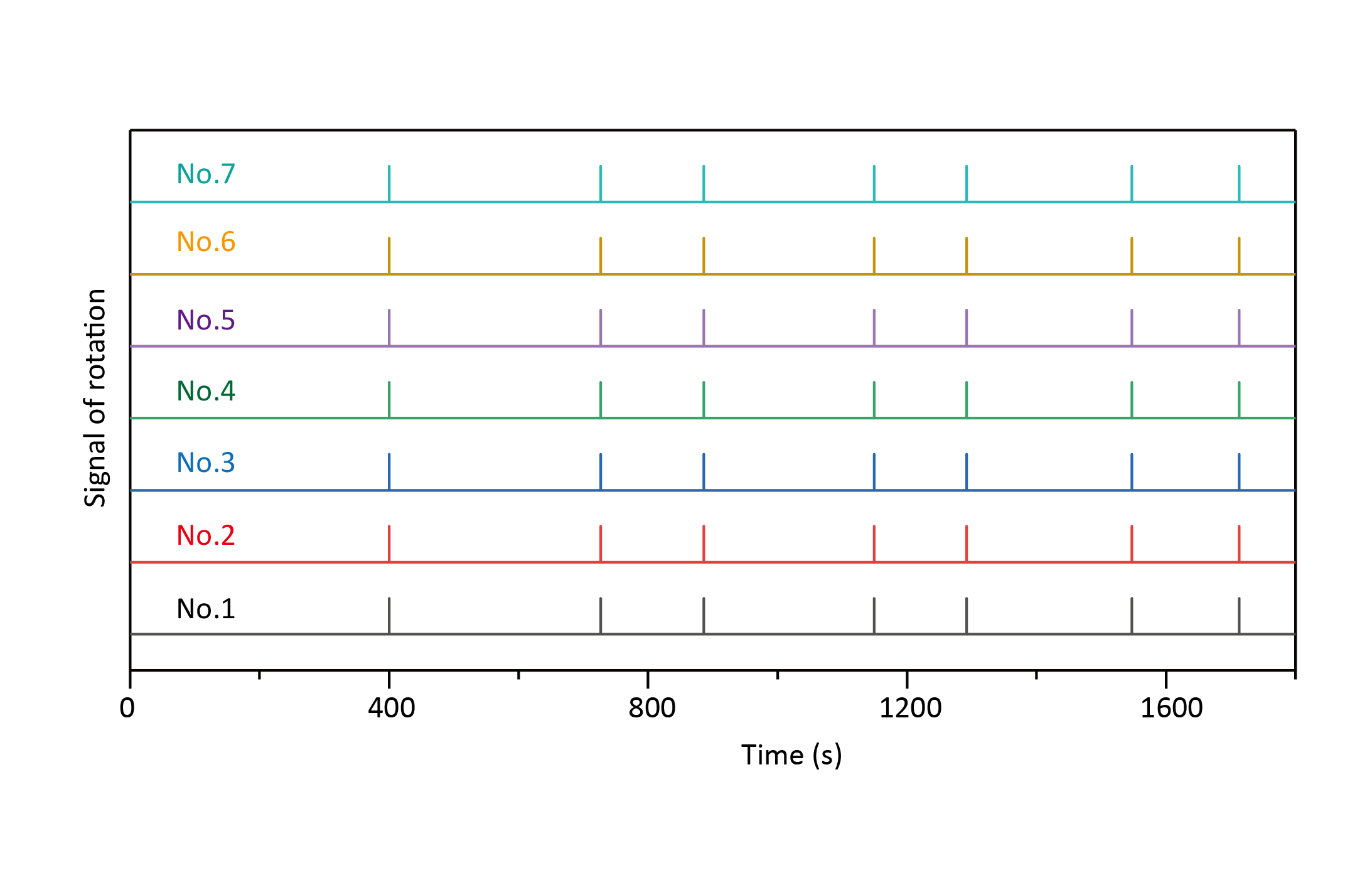


**Figure S9.** Rotations (as signals) for the heptamer school under 2-sun illumination.

*
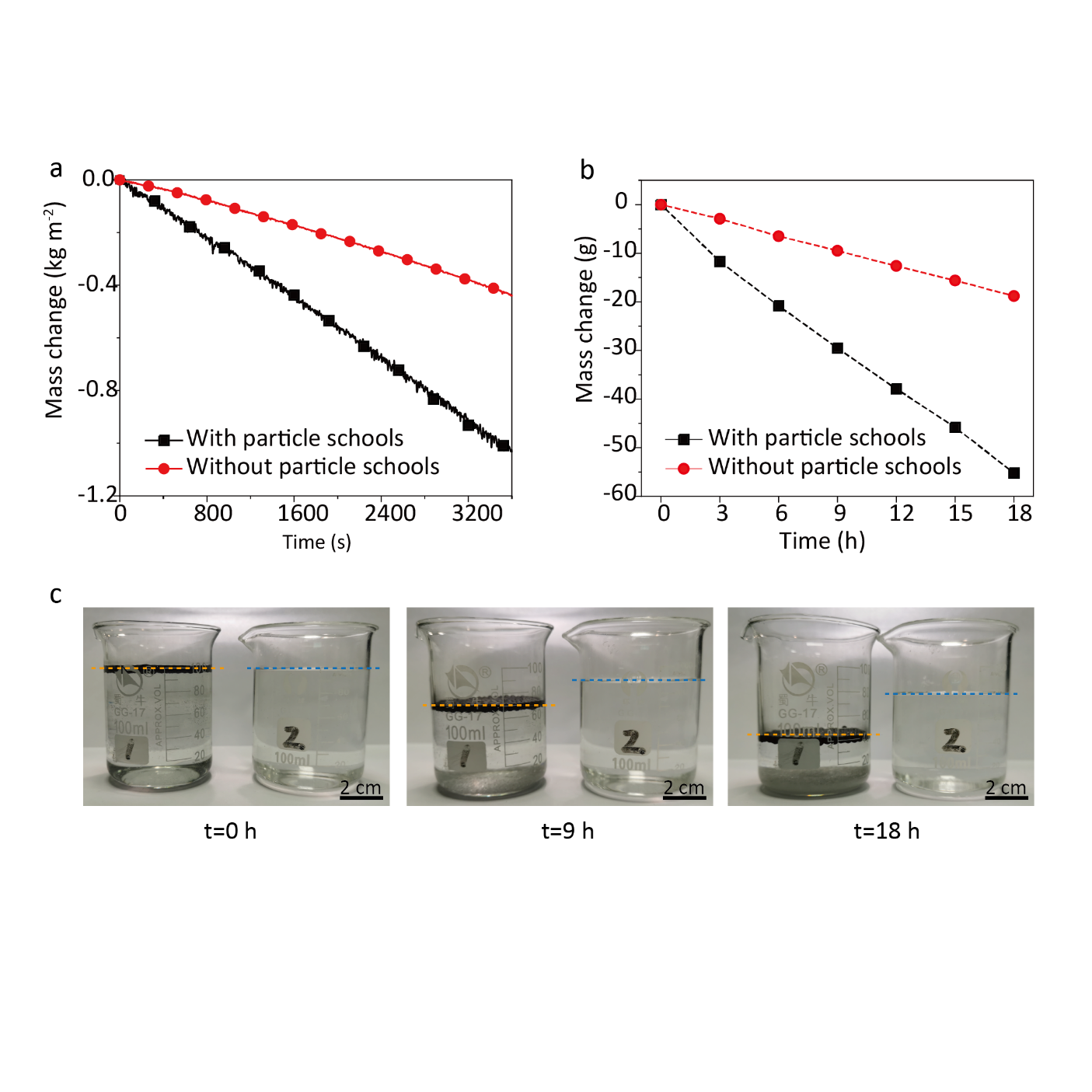
*

**Figure S10**. Performance comparison of saturated brine treatment with and without particle schools. (a) Mass change curves of saturated brine with and without particle schools under 1-sun illumination with thermal insulation (in Dewar). It shows that the saturated brine with particle schools can be treated much faster under sunlight illumination. (b) Mass changes over time of brine (initial salinity of ~25%) with and without particle schools under 2-sun illumination without thermal insulation (in beaker, for better observing the descending liquid surface). As the experiment is conducted under the simulated sunlight, which has a distance (between the light source and the sample surface) dependent intensity in the vertical direction, the light intensity on particle surfaces is decreased with the descending of the sample surface. After 18 h illumination, the illumination intensity on the particle surface (the brine with particle schools) is decreased to 1.67-sun, and that on the brine surface (the brine without particle schools) is 1.86-sun. Thus, it is observed a slight decrement of mass change rate in brine with particle schools. (c) The photos of the descending liquid levels for brine with and without particles. The yellow and blue dash lines indicate the surfaces of the brine with and without particle schools.


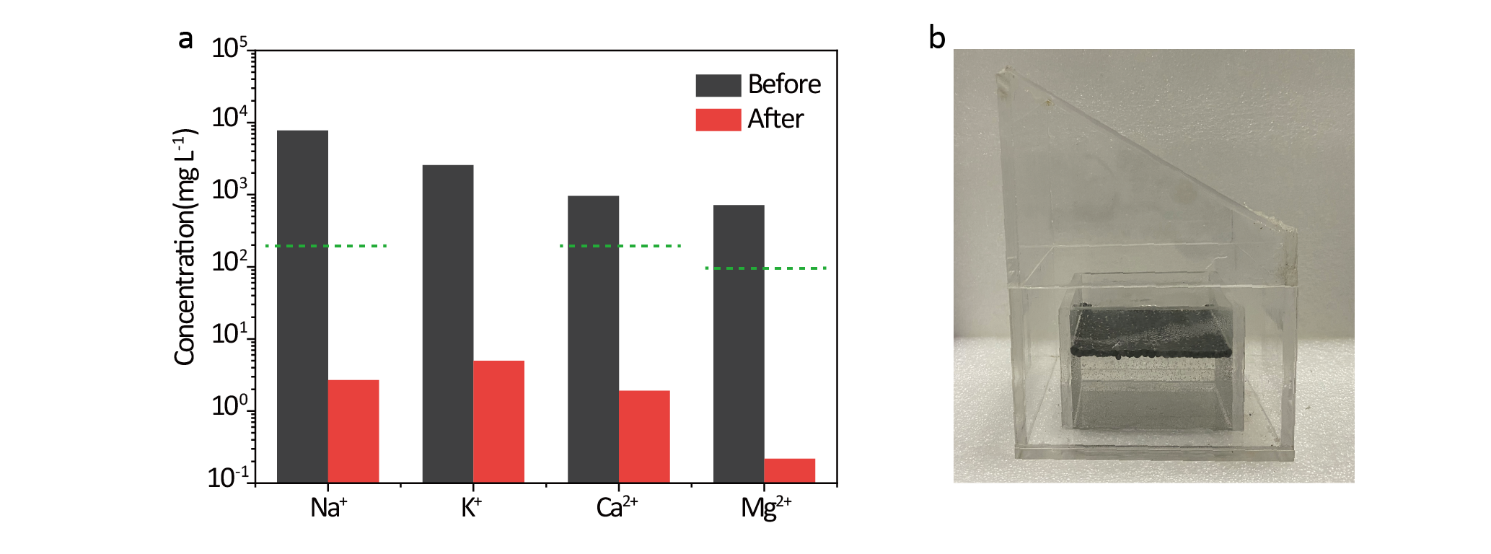


**Figure S11.** Ion concentrations of seawater and collected water, as well as the photo of the water collection device. (a) Ion concentrations in seawater and the vapor after solar desalination. Seawater with an average salinity of ~1 wt% (collected from the Bohai Sea, China) is used as the water source. It shows that the discharged vapor can meet the WHO standard (dashed green lines) [2]. (b) Water collection device with a collection efficiency of ~27% for getting the condensed vapor.


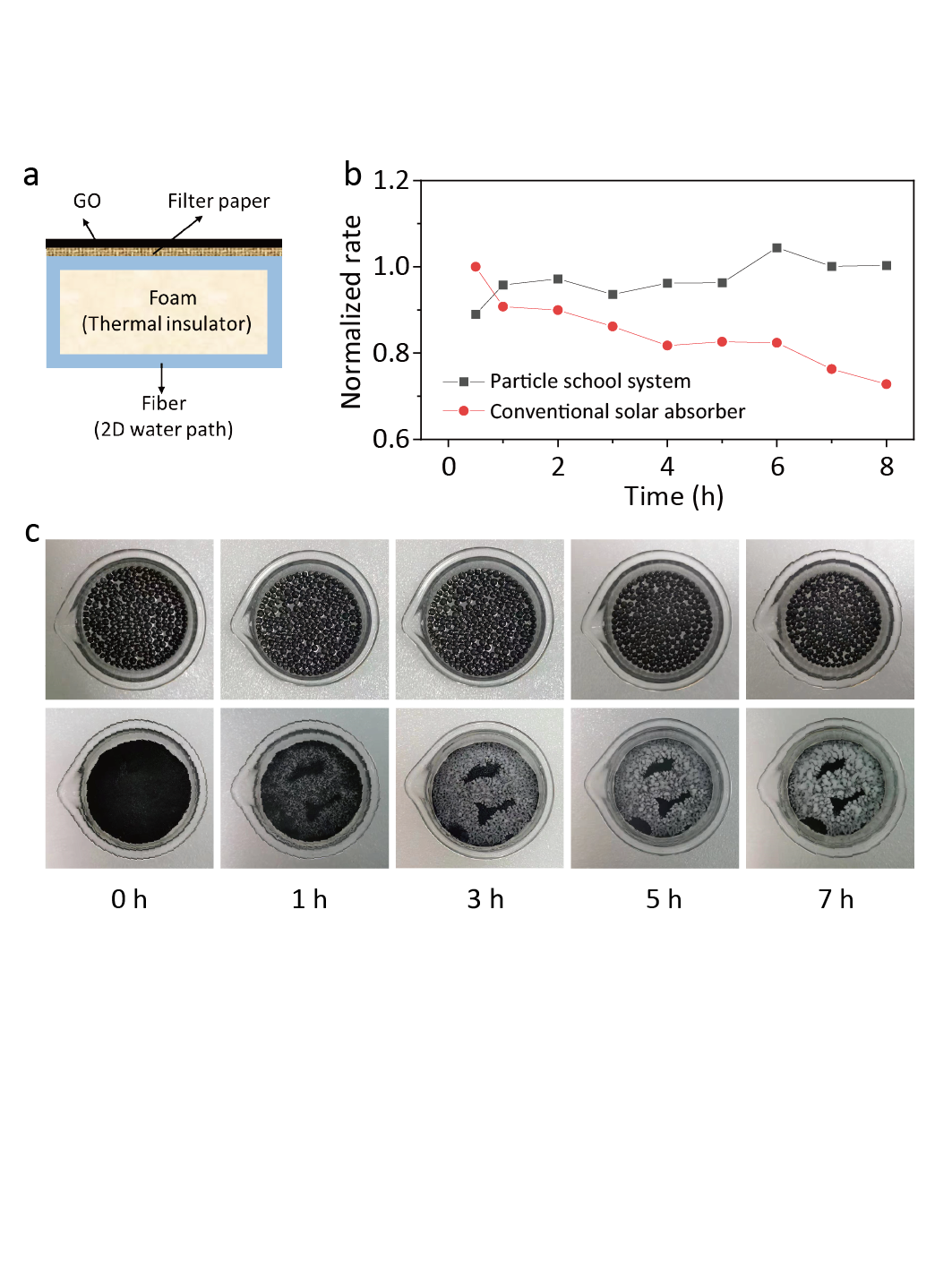


**Figure S12.** Long-term solar water treatment of the saturated brine for the particle school system and the conventional absorber. a, The schematic of the conventional absorber which is consisted of GO coated filter for solar absorption, fiber as the 2D water path, and the foam as the thermal insulator [3]. b, Normalized solar evaporation rates over time for the particle school system and the conventional solar absorber (normalized by the rate of the first half-hour of the conventional absorber). The solar evaporation rates of the particle systems keep stable during the long-term treatment, while that of the conventional solar absorber decreases gradually. c, Photos of the particle school system and the conventional absorber. The particle school system maintains clean during the long-term treatment, while salt aggregates on the surface of the conventional solar absorber, which blocks the sunlight and evaporation, leading to performance degradation.

**Table S1.** The real-time intensities of solar illumination for outdoor experiments.

| Date | Time | The intensity of solar illumination（W m^-2^） |
| --- | --- | --- |
| 2019/10/29 | 9 am | 372 |
| 2019/10/29 | 10 am | 527 |
| 2019/10/29 | 11 am | 605 |
| 2019/10/29 | 12 am | 623 |
| 2019/10/29 | 1 pm | 564 |
| 2019/10/29 | 2 pm | \| 462 \| \| --- \| |
| 2019/10/29 | 3 pm | 274 |
| 2019/10/29 | 4 pm | 116 |
| 2019/10/30 | 2 pm | 478 |
| 2019/10/30 | 3 pm | 328 |
| 2019/10/30 | 4 pm | 122 |
| 2019/10/31 | 9 am | 442 |
| 2019/10/31 | 10 am | 578 |
| 2019/10/31 | 11 am | 659 |
| 2019/10/31 | 12 am | 684 |
| 2019/10/31 | 1 pm | 632 |
| 2019/10/31 | 2 pm | 521 |
| 2019/10/31 | 3 pm | 365 |
| 2019/10/31 | 4 pm | 165 |
| 2019/11/1 | 9 am | 408 |
| 2019/11/1 | 10 am | 503 |
| 2019/11/1 | 11 am | 615 |
| 2019/11/1 | 12 am | 691 |
| 2019/11/1 | 1 pm | 461 |

**Reference**

1. Wu L. *et al*. Highly efficient three-dimensional solar evaporator for high salinity desalination by localized crystallization. *Nat Commun* 2020, **11**, 521.
2. World Health Organization (WHO). Safe drinking water from desalination; 2011.
3. Li X. *et al.* Graphene oxide-based efficient and scalable solar desalination under one sun with a confined 2D water path. *P Natl Acad Sci USA* 2016, 113, 13953.
